# Dental Ethics

**Published:** 1898-08

**Authors:** F. J. Capon

**Affiliations:** Toronto, Ontario.


ARTICLE IV.

### DENTAL ETHICS.

DR. F. J. CAPON, TORONTO, ONTARIA.

Ethics is the doctrine of morality, that philosophy which treats of human duties, their grounds and obligations.

The first duty to yourselves and to your patrons should be to aim high, for a low aim in our profession is more criminal than an honest failure. Conscientiousness should predominate, direct and guide us in all our work

Without the resolution in your hearts to do good work, so long as your hands have motion in them, and to do it whether the issue be that you die or live, no life worthy the name will ever be possible to you, while in once forming the resolution that your work is to be well done, life is easily won, now and forever.

John Newton said: "Oft-times the hindrances that lie in the path of duty may be compared to the toll gates on our turnpike roads—they are kept shut till we are just on them, and then fly open, as it were, of themselves. And that is time enough. If they had been opened a week beforehand, we could not but have gone through at last."

In these materialistic days it is hard for many men to rise to a truly professional standard. The idea has crept in that the most successful man is he who accumulates the greatest wealth, regardless of methods. The difference between a tradesman and the really professional man is that the former makes the dollar his highest aim, the chief end; while the latter makes the services he can render to humanity his highest aim, the chief end.

I know the temptations to stray from professional rectitude are numerous and strenuous. When a young man is making a struggle for bread a dollar bill looks as large as a horse blanket.

If the young man has normal stamina, if he has world foresight, he will realize that in professional life honesty is in every sense the "best policy." But to the young dentist who sits in his office and waits patiently but vainly for the "footsteps on the stairs" of the coming patient, it must seem indeed hard that his neighbor, and, possibly, colleague, who advertises "Real painless dentistry," "full sets for three dollars," "free extraction," etc., has his office constantly crowded with patients, and perhaps the young wonders if it really pays to live up to the highest standards of professional morality.

A few years ago I felt it my duty to act as a missionary to save a young colleague of mine who had fallen into evil ways, for "money is the root of all evil." I asked him, "Why did you sacrifice professional pride for the mighty dollar?" He was a man of few words; he threw out his chest, took a haughty stand, thrusting both hands to the depth of his trouser pockets, then withdrew the right hand slowly, bringing with it the pocket lining, showing its impoverished condition, remarking at the same time: "This is my professional pocket," and then he brought forth from the left pocket a huge roll of bank notes and said, "This is my advertisement pocket."

Then, why not run out cheap and nasty work for quick returns? Why not extract for the dollar the teeth that rational practice would save? Why not do many things just a little "off-color" for the sake of coin? I can sow my "wild oats" now and come back to correct living and take things easy. To such a young man, when he asks your advice, it is your duty to impress on his mind that honesty and plucky persistence bring rich rewards.

The professional man would rather render his services in a professional manner and receive no fee, than to render them unprofessionally and receive money. This, to some, may seem ideal, and not to be found among the men of any profession to-day. Well, the proportion may be small, but in the proportion that these professional men are to be found, the profession is reaching its highest aim.

The tradesman, nor the "professional" advertising writer, cannot understand these things. They are above them.

The dental profession is beset with this temptation to advertise as other professions. There is more intimacy with the trade side. The two come closer together.

We are tempted by our patients to do unprofessional things. They wish to dictate the manner of operations. Some will claim the right to say what we shall do. If you aim for a professional plane, never lose your dignity, and command respect. You must have the will-power to assert your authority and the nerve to see a patient walk out of your office in search of a less scrupulous man. This stand may mean dollars lost at the time, but to return in the future with added respect and confidence.

Morality, moral purity and integrity of character are also necessary if we would win the confidence and respect of our patients. At this period of psychological study we are subjected to such searching scrutiny, we become so sensitive that a moral taint of any kind is quickly felt or detected at a glance, and an instantaneous aversion or repugnance is the consequence. Who has not experienced this feeling on coming in contact with some persons, though they may be comparative strangers? It is like that of the blind man when he feels the presence of a second person in the room.

How much more keenly must such aversion be intensified in the close personal relation that necessarily exists between the operator and the patient, if he is not morally pure!

Then dental ethics embraces the often and so aptly quoted "Cleanliness is next to godliness." The most scrupulous care and attention must be given to personal neatness in every detail, especially in regard to the hands, nails and breath.

The morning bath is as essential to the hygienic dentist as the sterilizer is to the instruments he uses in the mouth of his patients. The operating coat should be suitable in texture and color and always neat and clean. I favor white linen—when soiled it can be seen.

You should endeavor to have the office and operating room an atmosphere of immaculate neatness, and thorough ventilation should prevail. It is indispensable that no odor of medicaments be detected, either in the apartments or about the person of the operator. A waste cotton receiver will prevent the carpet under your feet from becoming saturated with odious combinations. Nothing but the cleanest napery and the most thoroughly cleansed, sterilized and polished instruments should be used, especial care always being given to the mouth mirror. As few as possible of the necessary instruments and appliances should be exposed to view, thus avoiding undue suggestion of the operation to the patient. With the constant multiplicity of equipments there is also a danger of the operating room having the appearance of a machine shop. One of the most helpful and essential factors of a well-appointed office—one that greatly facilitates the operations, that attends to the appointments and cares for the instruments, and gives tone to the whole place—is the neat, refined and well dressed lady assistant. In my estimation, she is an indispensable adjunct to our work.

Ethics require that an operator should have professional skill and ability. Nothing is of greater value and importance to the operator in this direction than the acquiring and possession of a gentle yet firm and sure touch. For what can weary and excite a patient more than the uncertain or unskilled handling of sharp instruments, where a sudden slip might lacerate the pulp or inflict a wound on the surrounding tissues? Much valuable time may be wasted by an unsystematic arrangement of the necessary instruments for daily use—or from an unfamiliarity with and an unskilled use of them—and also by an indecision in quickly selecting the one best suited for a particular case. A good rule is to have a few carefully selected instruments and to learn to use them well. The awkward, noisy and unskilled manipulation of the instruments not only causes fear and annoyance to the patient, but also cruelty. We are charged with cruelty on every side. "I dread to enter a dental office." "I like you socially and as a friend, but I hate your profession." "Why did you ever engage in such a cruel calling?" are familiar expressions.

So general is the charge, so universal the complaint that little children, who do not know the meaning of the word "dentist," have to be dragged across our thresholds, and with quivering lips, pallid with fear, breaths tremulant with dread, beseeching eyes often filled with tears, beg for mercy before they reach the chair. You all know what I say is true; you hear it in society, at the club; we are caricatured in theaters, written about in papers. It is the same cry everywhere. We should try to obviate this cruel pain by administering anesthetics, local or general, by being gentle in our touch, soothing in our words, using the power of suggestion which will create a wonderful influence on a nervous patient. What pain is severer during the period of its duration than that endured in the extraction of a tooth? What pang more unendurable than that caused by the thrust of steel on an inflamed pulp? What will send shivers down one's back faster and colder than burring hypersensitive dentin? What will bring profanity to the lips of real gentlemen and tears to the eyes of women sooner than the grinding of carborundum wheels in the preparation of teeth for crowns? Who likes the prod of the broach against a root's pulp?

There is not a thoughtful man but knows that thousands of teeth are sacrificed daily in "painless dental parlors" and tens of thousands of dollars kept from the pockets of conscientious, reputable dentists, because of the dread of such suffering as I have described.

Tact, in my opinion, is an important factor in meeting our patients, and in successfully allaying any fear or apprehension that naturally arises with the patient. A quiet, reassuring demeanor, and a few sympathetic words, help greatly to accomplish this object; while nervous movements, brusque manners, harsh or careless expression, tend—especially with women and children—to create distrust and antagonism, and will repel the patient.

Another important but delicate point to emphasize is the need of our patients to understand the necessity of constant care of the mouth, the important and constant care of our little tender patients with their troublesome deciduous teeth.

In caring for temporary teeth, the heart should be full of love; in managing the permanent ones, the head should be full of wisdom.

The late Sir Benjamin Richardson, of England, thinks: "That the normal period of human life is about 110 years, and that seven out of ten people could live that long, if they lived in the right way. They should cultivate a spirit of serene cheerfulness under all circumstances, and should learn to like physical exercise in a scientific way. A happy disposition, plenty of sleep, a temperate gratification of all the natural appetites, and the right kind of physical exercise will ensure longevity to most people."

Practicing dentistry, like most indoor occupations, has its detrimental effects; it tends toward short life instead of longevity; and it becomes our duty to counteract the injurious effects that our calling produces. In a general way, we may say that dentists look pale, nervous, dyspeptic; have an enfeebled circulation; acquire a contracted chest, etc. When we take into consideration our contracted positions at the chair, the inhaling of poisoning exhalations of our patients, as well as the general nervous strained condition that we are in while doing delicate work, and all the time trying not to hurt, it is not strange that these should produce an enfeebled condition of health. When we ponder this fact, we must see at a glance the desirability, yes, necessity, of taking regular out-of door exercise.

For years I have made it a point to work in a gymnasium to keep an erect position, and have endeavored to be out of doors at least two hours of the twenty-four, either walking, riding horseback, bicycling, curling, canoeing or yachting—in fact, I am held to be a genuine sport.

Though I have spoken of good health last, I believe it is of almost the first importance, and in recapitulation might say that some of the important essentials of a successful practitioner are, first, health; second, tact; third, professional skill, conscientiousness and ability; fourth, integrity and moral and personal purity; fifth, a high aim.

*Odontoblast.*

